# Comparing Sarcopenia Definitions and Muscle Power Reduction: Associations with Health Outcomes in Spanish Community-Dwelling Older Adults

**DOI:** 10.3390/jcm13174988

**Published:** 2024-08-23

**Authors:** Juan Diego Ruiz-Cárdenas, Juan José Rodríguez-Juan, María del Mar Martínez-García, Alessio Montemurro

**Affiliations:** 1 Physiotherapy Department, Faculty of Physiotherapy, Podiatry and Occupational Therapy, Universidad Católica de Murcia, 30120 Murcia, Spain; mdmmartinez@ucam.edu (M.d.M.M.-G.); amontemurro@ucam.edu (A.M.); 2 Physiotherapy Department, Facultad de Medicina, Universidad de Murcia, Campus de Ciencias de la Salud, 30120 Murcia, Spain; jj.rodriguezjuan@um.es; 3 Cystic Fibrosis Association of Murcia, Av. de las Palmeras, 37, 30120 Murcia, Spain

**Keywords:** aging, sarcopenia, muscle power, risk factors, prevalence, smartphone

## Abstract

**Objectives**: To analyze the associations between the different operational definitions of sarcopenia published in the last decade and reduced muscle power with a set of adverse health-related outcomes, such as comorbidities, depression, polypharmacy, self-perceived health, educational attainment, socioeconomic status, falls, and hospitalizations in Spanish community-dwelling older adults. **Methods**: A total of 686 community-dwelling older adults (median age: 72; women: 59.2%; physically active: 84%) were included in this cross-sectional analysis (ClinicalTrials.gov: NCT05148351). Sarcopenia was assessed using the FNIH, EWGSOP2, AWGS, and SDOC algorithms. Reduced muscle power was defined as the lowest sex-specific tertile and measured during the rising phase of the sit-to-stand test using a validated mobile application. Unadjusted and adjusted logistic regressions by potential confounders were performed to identify the association between sarcopenia and reduced muscle power with health-related outcomes. **Results**: Sarcopenia prevalence was 3.4%, 3.8%, 12.4%, and 21.3% according to the SDOC, FNIH, EWGSOP2, and AWGS, respectively. Among these definitions, moderate and large associations with health-related outcomes were observed for EWGSOP2 and SDOC, respectively, but few associations were found for FNIH and AWGS criteria. Reduced muscle power was associated more frequently and moderately with health-related outcomes compared to sarcopenia definitions. These associations remained constant after adjusting for confounders. **Conclusions**: The prevalence and impact of sarcopenia varied depending on the definitions used. Among the sarcopenia definitions, the SDOC exhibited the strongest associations, while reduced muscle power was the variable most frequently associated with health-related outcomes compared to any of the four sarcopenia definitions in well-functioning and physically active community-dwelling older adults.

## 1. Introduction

Sarcopenia is an age-related muscle disease characterized by reduced muscle mass and strength or function, which can lead to negative health outcomes [1]. This condition has been associated with depression, comorbidities, polypharmacy, poor self-perceived health, and low socioeconomic status and educational attainment [2,3,4]. Sarcopenia also increases the risk of falls, hospitalizations, functional disability, and premature death [5,6].

The definition of sarcopenia has evolved over time. Initially defined by Rosenberg in 1989 as a reduction in lean body mass that affects ambulation, mobility, and independence [7], sarcopenia was more recently defined in 2020 by The Position Statements of the Sarcopenia Definition and Outcomes Consortium (SDOC) as a reduction in muscle strength and physical performance, without considering lean body mass [8]. Over the past decade, four different consensuses have been published: The Foundation for the National Institutes of Health (FNIH) [9], The European Working Group on Sarcopenia in Older People-2 (EWGSOP2) [10], The Asian Working Group for Sarcopenia (AWGS) [11], and the SDOC [8]. These definitions include simplified algorithms with specific cut-off points to facilitate the diagnosis of sarcopenia in research and clinical practice. However, the prevalence and impact of sarcopenia are largely determined by the diagnostic criteria used, as well as the characteristics of the population being studied. For example, the prevalence is higher among patients and those living in long-term care institutions compared to those living in the community [12,13] and ranges from 10% to 27% globally depending on the diagnostic criteria used [14].

The decline in muscle strength and mass that occurs with physiological aging is accompanied by a progressive reduction in muscle power, defined as the product of force and velocity. Although muscle power has never been included as a component of sarcopenia, the importance of its assessment in older adults is widely documented. Muscle power has been associated with several measures of physical function and sarcopenia components in community-dwelling older adults [15,16,17] and has been recognized as a better predictor of physical functioning and mortality compared to muscle strength [18,19]. Muscle power decreases with age more quickly and earlier than muscle mass and strength [19,20], which are the foundational factors in the definition of sarcopenia. Therefore, reduced muscle power might manifest early and be more closely associated with health-related outcomes than sarcopenia, highlighting the importance of its early assessment in community-dwelling older adults.

To our knowledge, only two studies have compared the associations between sarcopenia (as defined by the EWGSOP2) and reduced muscle power with adverse health-related outcomes [21,22]. In both studies, reduced muscle power showed greater clinical relevance than sarcopenia due to its associations with disability, physical performance, and frailty measures. However, the prevalence of sarcopenia in these studies was either not reported [22] or very small (n = 3, 0.8%) [21], which could compromise their findings. Moreover, since only one consensus was examined, these results might not be generalizable to other operational definitions. Lastly, the samples analyzed in these studies may exhibit lower physical function and greater disability compared to a more physically active population of older adults, affecting the risk estimated [23].

Therefore, the purpose of this study was to analyze the associations between the different operational definitions of sarcopenia published in the last decade (FNIH, EWGSOP2, AWGS, and SDOC) and reduced muscle power with a set of adverse health-related outcomes, such as comorbidities, depression, polypharmacy, self-perceived health, educational attainment, socioeconomic status, falls, and hospitalizations, in community-dwelling older adults attending elderly social centers where social and physical activities are promoted.

## 2. Materials and Methods

### 2.1. Study Design

This cross-sectional study is a secondary analysis from a previously published protocol [24] registered at ClinicalTrials.gov (registration number NCT05148351). The study followed the Declaration of Helsinki and received approval from the Ethical Committee of the Catholic University of Murcia (CE022108). The study was conducted in 11 elderly social centers of the city of Murcia (Spain) from February to October 2022. Primary outcomes were muscle power generated during a single sit-to-stand test and sarcopenia status assessed by four international consensuses (FNIH, EWGSOP2, AWGS, and SDOC). The secondary outcome was a clinical profile, which included health-related risk factors evaluated through a face-to-face interview (see below).

### 2.2. Eligibility Criteria

Older adults (≥60 years) who visited the elderly social centers were approached either by telephone or in person to receive information about participating in the study. Exclusion criteria were those participants at risk of dementia (Mini-Cog < 3 points), self-reporting cardiovascular problems (automatic defibrillator, pacemakers, heart valve disease, or uncontrolled heart rhythm problems), or those who reported problems or discomfort during the physical measures, e.g., participants who could not stand up from a chair without help or those who reported pain. Physical activity was measured with the Spanish Short Version of the Minnesota Leisure Time Physical Activity Questionnaire. Written informed consent was obtained from each participant in advance.

### 2.3. Procedures

The following tests were performed by the participants on the same day: sarcopenia assessment, the clinical interview, and the single sit-to-stand test.

#### 2.3.1. Sarcopenia Assessment

**Figure 1:**
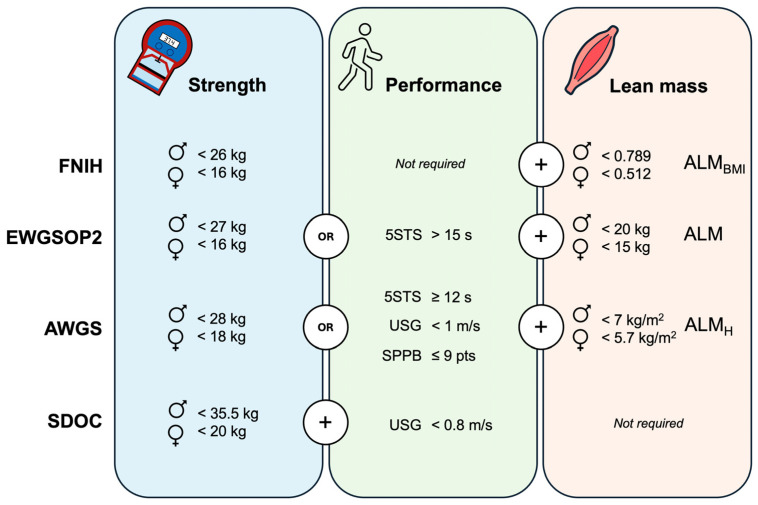
Cut-off points used for sarcopenia diagnosis according to the international consensuses. FNIH: The Foundation for the National Institutes of Health; EWGSOP2: The European Working Group on Sarcopenia in Older People-2; AWGS: The Asian Working Group for Sarcopenia; SDOC: The Position Statements of the Sarcopenia Definition and Outcomes Consortium; 5STS: five-chair stand test; SPPB: Short Physical Performance Battery; USG: usual gait speed; ♂: men; ♀: women; ALM: appendicular lean mass; ALM_H_: ALM relative to squared height; ALM_BMI_: ALM relative to body mass index. Muscle strength is assessed through handgrip testing.

The following tests were used for sarcopenia assessment according to four international consensuses. The handgrip strength test was performed using a digital dynamometer (Takei 5401, Takei Scientific Instruments Co., Ltd., Tokyo, Japan), with the participants seated with their forearms in a neutral position, resting flat on the chair arms, and their elbow flexed at 90 degrees. They were instructed to squeeze as hard as possible two times with each hand and the maximal score was recorded to determine reduced handgrip strength. The five-chair stand test was conducted on a standard-height chair. The participants were instructed to perform five complete repetitions as fast as possible from the sitting position with their arms crossed over their chest and the time was recorded using a stopwatch. The usual gait speed was collected using a 4 m walking test. The participants were instructed to walk at their normal pace. Two trials were recorded using a stopwatch and the best trial was used to determine reduced gait speed expressed in m/s. The Short Physical Performance Battery includes the measure of gait speed, balance, and the five-chair stand test. Briefly, gait speed was assessed as previously described. Balance was evaluated by examining the ability to stand with the feet together in the side-by-side, semi-tandem, and tandem positions for 10 s. The five-chair stand test was performed as previously described, but the stopwatch was stopped when the participant achieved the standing position at the end of the fifth stand [25]. Finally, appendicular lean mass (ALM) was estimated using resistance index and reactance values from bioelectrical impedance analysis (TANITA MC-580, Tanita Corp., Tokyo, Japan). These values were introduced in Sergi’s validated equation to estimate ALM [26]. Then, sarcopenia prevalence was calculated following the specific cut-off points provided by the international consensuses (Figure 1).

#### 2.3.2. Clinical Profile

Self-reported health-related outcomes were recorded through face-to-face interviews. Participants were interviewed regarding the presence of depressive symptoms using the Geriatric Depression Scale Spanish version 5-items (cut-off point: ≥2); socioeconomic status (cut-off point: net salary < 10,000 EUR/year); educational attainment (cut-off point: primary or less); presence of comorbidity (≥2 chronic conditions); polypharmacy (≥5 drugs/day); self-perceived health (cut-off point: very bad, bad, or fair); presence of two or more falls in the last year, defined as an unexpected event in which the individual came to rest on the ground, floor, or lower level [27]; and hospitalizations in the last year. Further details on the recorded health-related outcomes are available in the study protocol [24].

#### 2.3.3. Single Sit-to-Stand Muscle Power Test

The *Sit to Stand* app (v. 2.0.2) installed on an iPhone 13 device running iOS 15.3 was used to estimate muscle power during a single sit-to-stand test. This app was designed to analyze the rising phase of the sit-to-stand movement via high-speed video recording at 240 frames per second. Participants, seated on an adjustable-height chair without footwear, were instructed to stand up as quickly as possible with their arms crossed over their chest and the hip, knee, and ankle joints at 90 degrees. The test was recorded with the smartphone positioned horizontally on a 0.7 m high tripod placed 3 m from the right or left side of the participants following the app’s instructions. Vertical muscle power (W/kg) was estimated from the app based on the following regression equation (*R^2^* adjusted = 0.917; *p* = 0.035; standard error of estimate = 0.45):

Power (W/kg) = 2.773 − 6.228 × *t* + 18.224 × *d*

where *t* represents the rising time as measured by selecting two frames during the video analysis and *d* is the vertical displacement, which is matched with the participant’s femur length when they are sat with their knee at a right angle. The participant’s femur length was measured as the distance between the greater trochanter and the lateral condyle using a metric tape. Three trials were performed with 30 s of rest between trials and the mean value was used for further analyses. Muscle power derived from this app has been previously validated against force platforms and 3D motion capture cameras in adults with a broad age range (21–91 years) [16,28]. Reduced muscle power was defined as the lowest sex-specific tertile.

### 2.4. Data Analysis from the Sit to Stand App

**Figure 2:**
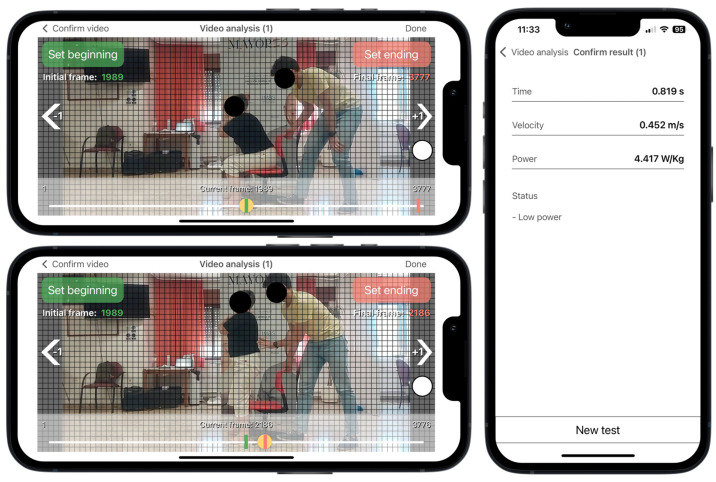
*Sit to Stand* app user interface running on an iPhone 13. Video analysis at 240 frames per second selecting the first frame corresponding to the beginning of the rising phase, when the red dot crosses the first horizontal grid line (horizontal top panel), and the end of the rising phase, when the red dot achieves the highest vertical point (horizontal lower panel). Results from the app after video analysis (vertical panel). Example for a non-sarcopenic women with reduced muscle power and femur length of 0.37 m. Red dot represents the colored sticker placed on the greater trochanter.

According to the app’s instructions, the rater selected two videoframes corresponding with the onset and the end of the rising phase. Prior to the execution of the test, a visual sticker was placed on the superior aspect of the greater trochanter for helping the rater to detect the beginning and the end of the rising phase during the video analysis (Figure 2). The onset of the rising phase was defined as when the sticker crossed the first horizontal grid line, which was time-matched with when the pelvis began to move forward after anterior trunk tilt. The rising phase ended when the sticker achieved the highest vertical point, which was time-matched with when full extension of the hip and knee was achieved in an upright stance. The app has shown excellent inter-rater and intra-rater reliability for measuring muscle power (intra-class correlation coefficient > 0.97) [17].

### 2.5. Statistical Analysis

Statistical analyses were performed using IBM SPSS Statistics 26.0 (SPSS Inc. Chicago, IL, USA). Continuous variables were presented as means and standard deviations if normally distributed, otherwise as medians and interquartile ranges. Frequencies and percentages were used for categorical data. To examine the association between sarcopenia and muscle power with health-related outcomes, binary logistic regressions were performed with non-sarcopenic and normal power as the referent group. Models were first performed unadjusted, and then, adjusted for potential confounders (age, sex, and body mass index). The odds ratio (OR) was calculated and interpreted as small (<1.7), moderate (1.7 to 3.5), and large (>3.5) associations [29]. Statistical significance was fixed at *p* <0.05.

## 3. Results

### 3.1. Participant Characteristics

A total of 686 community-dwelling older adults (59.2% women) aged between 60 and 88 years, with 84% physically active, were included in this cross-sectional analysis. Of those, 129 (18.8%) had depressive symptoms, 133 (19.4%) reported poor self-perceived health, and 145 (21.1%) were taking five or more prescription drugs per day. A total of 527 (76.8%) participants had the presence of two or more comorbidities, 85 (12.4%) had suffered two or more falls in the past year, and 90 (13.1%) had been hospitalized at least once in the past year. The educational attainment and socioeconomical status were low for 319 (46.5%) and 97 (14.1%) participants, respectively.

The prevalence of sarcopenia was lowest according to the SDOC criteria, with 23 participants (3.4%), followed by the FNIH criteria with 26 participants (3.8%), the EWGSOP2 criteria with 85 participants (12.4%), and the AWGS criteria with 146 participants (21.3%). A total of 228 (33%) participants were categorized with reduced muscle power according to the lowest sex-specific tertile (<5.61 W/kg in men and <4.69 W/kg in women) (Table 1).

### 3.2. Associations between Sarcopenia and Muscle Power with Health-Related Outcomes

Sarcopenic individuals diagnosed by the SDOC showed the strongest associations with health-related outcomes among the sarcopenia criteria analyzed in this study. Sarcopenic individuals according to the SDOC showed large associations with low educational attainment (OR: 5.6; 95% CI: 1.9 to 16.5), polypharmacy (OR: 11.8; 95% CI: 4.5 to 30.5), poor self-perceived health (OR: 4.1; 95% CI: 1.7 to 9.4), and hospitalizations (OR: 3.1; 95% CI: 1.2 to 7.6). These associations remained constant, although lower in magnitude, after adjusting for potential confounders. Sarcopenic individuals diagnosed by the FNIH criteria showed moderate-to-large associations with low educational attainment, comorbidities, polypharmacy, poor self-perceived health, and depression. However, after adjusting for potential confounders only self-perceived health was associated with sarcopenia according to the FNIH (OR: 3.2; 95% CI: 1.4 to 7.6). No associations were found in a univariate analysis for those sarcopenic individuals diagnosed by the AWGS. However, after adjusting for confounders, sarcopenic individuals showed small-to-moderate associations with low educational attainment (OR: 1.9; 95% CI: 1.3 to 2.9), comorbidities (OR: 1.6; 95% CI: 1.1 to 2.7), and falls (OR: 2.1; 95% CI: 1.2 to 3.8). The EWGSOP2 was the sarcopenia consensus most associated with health-related outcomes. Sarcopenic individuals diagnosed by the EWGSOP2 showed moderate associations with low educational attainment (OR: 1.8; 95% CI: 1.1 to 2.8), comorbidities (OR: 2.5; 95% CI: 1.2 to 4.9), polypharmacy (OR: 2.4; 95% CI: 1.1 to 2.8), poor self-perceived health (OR: 2.5; 95% CI: 1.5 to 4.1), depression (OR: 1.9; 95% CI: 1.2 to 3.3), and hospitalizations (OR: 2.2; 95% CI: 1.2 to 3.8). These associations remained stable after adjusting for potential confounders except for low educational attainment (*p* > 0.05).

Reduced muscle power was the variable most frequently associated with health-related outcomes compared to the four sarcopenia definitions. Participants categorized with reduced muscle power showed moderate associations with low educational attainment (OR: 1.9; 95% CI: 1.4 to 2.6), low income (OR: 1.8; 95% CI: 1.1 to 2.8), comorbidities (OR: 2.5; 95% CI: 1.6 to 3.8), polypharmacy (OR: 1.9; 95% CI: 1.4 to 2.9), poor self-perceived health (OR: 2.7; 95% CI: 1.8 to 3.9), depression (OR: 1.8; 95% CI: 1.2 to 2.6), and falls (OR: 1.8; 95% CI: 1.1 to 2.9). The associations remained stable after adjusting for confounders, but slightly lower in magnitude, except for low income (*p* > 0.05) (Table 2).

## 4. Discussion

This study compared the associations between different operational definitions of sarcopenia published in the past decade (FNIH, EWGSOP2, AWGS, and SDOC) and reduced muscle power with a range of adverse health-related outcomes. Among these definitions, the SDOC consensus exhibited the strongest associations, while reduced muscle power was more frequently associated with these outcomes than any of the four sarcopenia definitions in physically active, community-dwelling older adults with good overall health and physical performance.

The lowest prevalence of sarcopenia was found using the FNIH (3.8%) and SDOC (3.4%) definitions. While the FNIH was only associated with poor self-perceived health, the SDOC additionally showed large associations with low educational attainment, polypharmacy, and hospitalizations. Similarly, the EWGSOP2 definition demonstrated moderate associations with comorbidities, depression, poor self-perceived health, polypharmacy, and hospitalizations. These findings are in line with a recent scoping review that analyzed the predictive validity of current sarcopenia definitions for health-related adverse events in community-dwelling older adults [30]. Despite the inability to perform a meta-analysis due to significant methodological heterogeneity among the included studies, the FNIH operational definition was not predictive of mortality, institutionalization, physical disability, falls, or fractures compared to other sarcopenia definitions such as the SDOC and EWGSOP2. Consistent with our study, the SDOC showed the strongest associations, but in this case for all types of fractures analyzed. However, any study included in this scoping review analyzed the predictive validity of the AWGS operational definition [30]. In our study, AWGS showed small-to-moderate associations with low educational attainment, comorbidities, and falls, but only when adjusted for confounders. In contrast, the associations found for EWGSOP2 and SDOC remained constant in both unadjusted and adjusted models, highlighting the validity of these operational definitions. Moreover, these definitions were the only ones associated with hospitalizations.

Although the SDOC showed the strongest associations in our study, it also reported the lowest prevalence. Despite the SDOC proposing the highest sensitivity cut-off points for muscle strength among all sarcopenia definitions (Figure 1), the cut-off point for slowness is relatively low (<0.8 m/s) and rarely observed in well-functioning, physically active older adults [23,31]. These factors may account for the low prevalence rate found in our sample, which consisted predominantly of physically active individuals. The prevalence found in our study using the EWGSOP2 algorithm was 12.4%, which aligns with the global prevalence estimate of 10% (95% CI: 2.0 to 17) in older adults from a recent meta-analysis [14]. However, the highest prevalence rate (21.3%) was observed using the AWGS definition, probably due to its revised criteria. While the estimated prevalence among community-dwelling older adults using the 2014-AWGS version is 12.9% [32], the AWGS revised version used in our study incorporates higher sensitivity cut-off points for muscle strength and gait speed, as well as additional physical performance tests like the five-chair stand test and the Short Physical Performance Battery, which were not included in the previous version [33]. This likely accounts for the increased prevalence rate found in our study.

As well documented in previous studies and confirmed by our findings, the prevalence and impact of sarcopenia largely depend on the diagnostic criteria used. The lack of a single operational definition remains a major obstacle for identifying and treating people with sarcopenia and understanding its impact. For example, the incidence, prevalence, economic burden, and even consequences of sarcopenia are often difficult to summarize because different definitions result in significant discrepancies or make reliable estimates unfeasible [14,30,34]. Additionally, a person might be diagnosed or undiagnosed depending on the cut-off point used [4,35], causing uncertainty for assessors and clinicians [1]. Efforts are being made to establish a global consensus for sarcopenia diagnosis to address these issues. The Global Leadership Initiative in Sarcopenia (GLIS) recently published a Delphi consensus to harmonize these competing definitions into one standard for sarcopenia assessment [1]. The expert panel agreed to define sarcopenia as the combination of reduced muscle strength and mass, while physical performance was accepted as an outcome rather than a component of sarcopenia. In our study, the FNIH was the only consensus following the conceptual definition of sarcopenia proposed by the GLIS, which excludes physical performance as a component. However, it showed the fewest associations with adverse health-related outcomes. Since the diagnostic criteria for sarcopenia should be determined by their ability to predict hard outcomes, these findings call into question the cut-off points used by the FNIH in well-functioning community-dwelling older adults and should be considered in the following algorithm proposed by the GLIS consensus.

Despite the importance of assessing muscle power in older adults, the expert panel of the GLIS did not agree to include muscle power in the conceptual definition of sarcopenia, with this statement receiving the lowest agreement (~68%) [1]. Furthermore, this measure was not included in a recent consensus by the European Society for Clinical and Economic Aspects of Osteoporosis, Osteoarthritis, and Musculoskeletal Diseases, which aimed to identify the most applicable tests for assessing muscle function and physical performance in older adults in daily clinical practice [36]. The primary reason for its exclusion was the difficulty of measuring muscle power in clinical settings, since it requires complex and expensive equipment, is time consuming, lacks standardized protocols and specific cut-off points, and necessitates training for both clinicians and subjects [36]. This mobile app could help overcome the aforementioned barriers by offering a free, easy-to-use tool for measuring muscle power in clinical settings, thus bridging the gap between research and clinical practice. Muscle power derived from this app has been associated with several measures of physical function and sarcopenia and frailty determinants in community-dwelling older adults [16,17,28]. Additionally, this app has recently been used to evaluate performance fatigability by detecting reductions in muscle power after both isometric and dynamic fatigue protocols in healthy adults [37,38]. Considering that age-related neuromuscular adaptations alter performance fatigability, this assessment may also serve as an early indicator of the onset of disability, frailty, and mortality in older adults [39,40]. Our study stablished a cut-off point of reduced muscle power as the lowest sex-specific tertile in a sample of community-dwelling older adults physically active and with a good health status overall. Participants categorized as reduced muscle power by the app showed greater odds of adverse health-related outcomes such as low educational attainment, comorbidities, polypharmacy, poor self-perceived health, and depression. Furthermore, muscle power was the only variable associated with previous falls in unadjusted models and remained largely consistent after adjusting for potential confounders, underscoring its validity.

The importance of assessing muscle power in older adults extends beyond its associations with functional mobility and health-related adverse events. Muscle power is often proposed as the primary therapeutic target for resistance training interventions in this population [41]. Additionally, muscle power assessed during the rising phase of a sit-to-stand test has shown greater responsiveness after an exercise training program compared with the total time to complete the test or other clinical measures, such as isometric quadriceps strength, the timed up and go test, and the Berg Balance Scale [42]. Since muscle power declines earlier and more rapidly than muscle strength and mass, it should be considered an important complementary measure in daily clinical practice, as it could potentially detect earlier functional impairment and health-related adverse outcomes than current sarcopenia measures. Considering that most health care professionals do not diagnose sarcopenia, mainly due to lack of equipment and time constraints [43,44,45], measuring muscle power using an affordable instrument could potentially reduce the incidence of adverse health-related consequences, inform about prognosis, and reduce health care cost. Moreover, engaging mobile applications enhance evaluation by providing positive experiences and boosting motivation. Interactive features and user-friendly interfaces could make evaluations enjoyable, fostering understanding and encouraging continued participation.

One of the main limitations of our study is the retrospective validation of the specific cut-off points provided by the app. Although these cut-off points were based on sex-specific tertiles in a physically active population with good health status and physical performance overall, their external validity needs further evaluation. Additionally, the relationships between sarcopenia definitions, muscle power, and health-related risk factors were based on self-reported data from a single time point. As a result, the information relied on the subjects’ perceptions, and cross-sectional data cannot establish causal relationships as these observations may reflect bidirectional associations.

## 5. Conclusions

The prevalence and impact of sarcopenia varied depending on the definitions used. Among the sarcopenia definitions, the SDOC exhibited the strongest associations, while reduced muscle power was most frequently associated with health-related outcomes compared to any of the four sarcopenia definitions in well-functioning and physically active, community-dwelling older adults.

## Figures and Tables

**Table 1:** Characteristics of the participants (N = 686).

Outcome	Women	Men
Number of participants (%)	406 (59.2)	280 (40.8)
Age (years)	71 (68–75)	72 (68–76)
Height (m)	1.55 (0.06)	1.68 (0.06)
Body mass (kg)	66 (59.6–73.2)	79.3 (71.6–88.5)
BMI (kg/m^2^)	27.4 (24.4–30.1)	28.1 (25.8–31.2)
ALM (kg)	14.4 (13.1–15.7)	20.2 (18.7–22.3)
ALM_height_ (kg/m^2^)	5.9 (5.5–6.4)	7.2 (6.7–7.7)
ALM_BMI_	0.52 (0.48–0.57)	0.72 (0.67–0.77)
Femur length (m)	0.36 (0.34–0.38)	0.39 (0.36–0.4)
Handgrip strength (kg)	22.6 (4.3)	37.5 (6.8)
5STS (s)	12.1 (10.6–14.1)	12.2 (10.7–14.2)
UGS (m/s)	1.19 (0.25)	1.19 (0.26)
SPPB (pt)	12 (11–12)	12 (11–12)
Muscle power (W/kg)	5.0 (1.1)	5.89 (1.0)
Physical activity levels (%)		
Sedentary	49 (12.1)	61 (21.8)
Physically active	357 (87.9)	219 (78.2)
Sarcopenia prevalence (%)		
FNIH	15 (3.7)	11 (3.9)
EWGSOP2	56 (13.8)	29 (10.4)
AWGS	85 (20.9)	61 (21.8)
SDOC	10 (2.5)	13 (4.6)
Reduced muscle power (%)	134 (33)	94 (33)

Continuous data are reported as mean and standard deviation or median and interquartile range, whereas categorical data are reported as frequency and percentages. BMI: body mass index; ALM: appendicular lean mass; ALM_height_: ALM relative to squared height; ALM_BMI_: ALM relative to BMI; 5STS: five-chair stand test; UGS: usual gait speed; SPPB: Short Physical Performance Battery; FNIH: The Foundation for the National Institutes of Health; EWGSOP2: The European Working Group on Sarcopenia in Older People-2; AWGS: The Asian Working Group for Sarcopenia; SDOC: The Position Statements of the Sarcopenia Definition and Outcomes Consortium (SDOC). Sedentary was defined as < 500 metabolic equivalent task (MET) min per week.

**Table 2:** Associations between sarcopenia and reduced muscle power with health-related outcomes (N = 686).

	FNIH		EWGSOP2		AWGS		SDOC		Reduced Muscle Power	
Health Outcome	OR (95% CI)	Adjusted	OR (95% CI)	Adjusted	OR (95% CI)	Adjusted	OR (95% CI)	Adjusted	OR (95% CI)	Adjusted
Low educational attainment (n, 319)	3.69 (1.46–9.37) *	1.95 (0.73–5.18)	1.76 (1.11–2.8) *	1.53 (0.92–2.56)	1.35 (0.93–1.95)	1.94 (1.27–2.98) *	5.57 (1.87–16.5) *	3.25 (1.05–10.1) *	1.89 (1.37–2.61) *	1.45 (1.02–2.04) *
Low income (n, 97)	1.93 (0.75–4.99)	1.15 (0.43–3.2)	1.46 (0.8–2.65)	1.23 (0.64–2.36)	1.23 (0.74–2.04)	1.58 (0.88–2.83)	1.69 (0.61–4.69)	1.06 (0.36–3.12)	1.77 (1.14–2.76) *	1.44 (0.9–2.3)
Comorbidities (n, 527)	7.87 (1.06–58.5) *	5.55 (0.72–42.8)	2.47 (1.25–4.91) *	2.33 (1.23–4.81) *	1.21 (0.77–1.9)	1.66 (1.01–2.73) *	2.05 (0.6–6.99)	1.61 (0.44–5.9)	2.46 (1.6–3.79) *	2.05 (1.3–3.22) *
Polypharmacy (n, 145)	2.42 (1.07–5.45) *	1.33 (0.55–3.2)	2.44 (1.5–3.96) *	2.55 (1.47–4.41) *	1.11 (0.71–1.72	1.55 (0.93–2.59)	11.8 (4.55–30.5) *	7.93 (2.92–21.5) *	1.97 (1.36–2.87) *	1.5 (1.1–2.24) *
Poor self-perceived health (n, 133)	4.5 (2.03–9.95) *	3.22 (1.37–7.58) *	2.47 (1.51–4.06) *	2.55 (1.46–4.44) *	1.04 (0.66–1.65)	1.43 (0.84–2.42)	4.06 (1.75–9.43) *	3.12 (1.26–7.23) *	2.67 (1.82–3.93) *	2.26 (1.5–3.4) *
Depression (n, 129)	2.84 (1.26–6.42) *	2.13 (0.89–5.1)	1.99 (1.19–3.32) *	1.84 (1.05–3.24) *	0.97 (0.61–1.56)	1.09 (0.64–1.85)	1.94 (0.78–4.82)	1.51 (0.57–3.95)	1.79 (1.2–2.64) *	1.6 (1.06–2.42) *
Falls (n, 85)	1.72 (0.63–4.69)	1.34 (0.46–3.89)	1.78 (0.98–3.24)	1.64 (0.85–3.17)	1.64 (0.99–2.73)	2.12 (1.17–3.83) *	1.51 (0.5–4.5)	1.3 (0.41–4.17)	1.84 (1.16–2.91) *	1.74 (1.1–2.83) *
Hospitalizations (n, 90)	1.61 (0.59–4.37)	1.5 (0.51–4.38)	2.18 (1.23–3.84) *	2.23 (1.19–4.18) *	1.62 (0.98–2.67)	1.49 (0.85–2.64)	3.05 (1.22–7.63) *	2.83 (1.05–7.64) *	1.13 (0.71–1.8)	1.18 (0.72–1.93)

Low educational attainment (primary or less), low income (net salary < 10,000 EUR/year), comorbidities (≥2 chronic conditions), polypharmacy (≥5 drugs/day), poor self-perceived health (very bad, bad, or fair), depression (GDS-5 ≥ 2), falls (≥2 last year), hospitalizations (≥1 last year). FNIH: The Foundation for the National Institutes of Health; EWGSOP2: The European Working Group on Sarcopenia in Older People-2; AWGS: The Asian Working Group for Sarcopenia; SDOC: The Position Statements of the Sarcopenia Definition and Outcomes Consortium. Reduced muscle power was defined as the lowest sex-specific tertile (<5.61 W/kg in men and <4.69 W/kg in women). Adjusted odds ratio (OR) for age, sex, and body mass index with its 95% confidence interval (CI). Statistical significance at an alpha level 0.05 is represented by asterisks (* *p* < 0.05).

## Data Availability

All data generated or analyzed during this study are included in this article. Datasets may be made available to editors, reviewers, and readers upon request to the corresponding author.
